# Mapping Atomic-Scale Metal–Molecule Interactions: Salient Feature Extraction through Autoencoding of Vibrational Spectroscopy Data

**DOI:** 10.1021/acs.jpclett.3c01483

**Published:** 2023-08-18

**Authors:** Alex Poppe, Jack Griffiths, Shu Hu, Jeremy J. Baumberg, Margarita Osadchy, Stuart Gibson, Bart de Nijs

**Affiliations:** † School of Physics and Astronomy, University of Kent, Canterbury CT2 7NH, U.K.; ‡ NanoPhotonics Centre, Cavendish Laboratory, University of Cambridge, Cambridge CB3 0HE, U.K.; § Computer Science Department, University of Haifa, Haifa 3498838, Israel

## Abstract

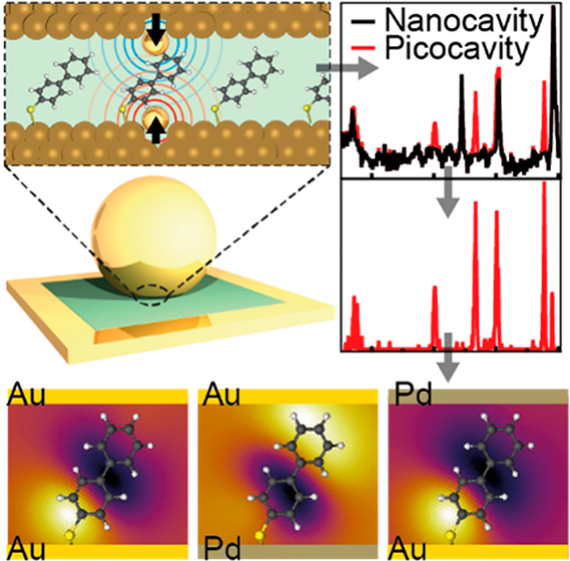

Atomic-scale features, such as step edges and adatoms, play key roles in metal–molecule interactions and are critically important in heterogeneous catalysis, molecular electronics, and sensing applications. However, the small size and often transient nature of atomic-scale structures make studying such interactions challenging. Here, by combining single-molecule surface-enhanced Raman spectroscopy with machine learning, spectra are extracted of perturbed molecules, revealing the formation dynamics of adatoms in gold and palladium metal surfaces. This provides unique insight into atomic-scale processes, allowing us to resolve where such metallic protrusions form and how they interact with nearby molecules. Our technique paves the way to tailor metal–molecule interactions on an atomic level and assists in rational heterogeneous catalyst design.

The pervasiveness of catalytic processes, a drive for materials efficiency, and the minimization of precious resource utilization have developed a need for resolving molecular interactions at heterogeneous interfaces at an atomic level.^1,2^ However, few methods offer this level of resolution, and none currently allow for in-operando studies. Promising techniques are emerging with submolecular sensitivity but heavily rely on indirect interpretation of spectroscopic data, making such processes prohibitively time-consuming to model.^3−5^ To this end, bespoke and robust analysis methods are required that can digest large data sets to build up a comprehensive understanding of the atomic scale processes involved.

Deep learning is a multifaceted data processing method with a range of applications in spectroscopy due to its ability to detect complex, often nonlinear features and process large quantities of data with high throughput. As a result, deep learning has provided powerful tools that can classify substances or predict quantities without the need for potentially bias-inducing preprocessing^6^ steps, which are commonly required in alternative methods such as partial least-squares regression. This attribute allows deep learning to process e.g. mixtures consisting of multiple chemical compounds^7^ or spectra with highly variable baselines. Such methods are also capable of providing identification despite a small number of reference samples (or even from individual reference spectra).^8^ This is because neural networks offer a robustness to variability in spectra that is not linked to the underlying information we aim to qualify. Thus, they are particularly effective at categorizing spectra pertaining to unique molecular compositions, states, or transitory physical events. Through pattern recognition, machine learning algorithms can extract salient features from unlabeled surface-enhanced Raman spectroscopy (SERS) data. These features may arise from chemical changes in the analyte molecules, when new molecules are introduced, or from morphological changes in the metal surface.^5,9−13^ Such features are particularly prevalent in SERS spectra of a few or single molecules. These are traditionally difficult to study due to their transient nature but offer in return a unique opportunity to elucidate the behavior of molecules on an atomic length scale.^3^ We note that the analysis of complex spectroscopic data remains an unsolved challenge in machine learning.

Here we show that a combination of machine learning and image processing techniques can serve as a tool to analyze the spatiotemporal properties of a collection of SERS data sets and demonstrate the potential to extend this technique to further spectroscopic data. We focus on spectral changes brought about by atomic-scale features (e.g., step edges and adatoms) forming in metal surfaces during irradiation with light. These undercoordinated metal sites provide binding sites for important and often desirable metal–molecule interactions, facilitating applications such as heterogeneous catalysis,^1,2,14,15^ molecular electronics,^16^ memristive switching,^17^ and ultrasensitive sensing.^3,18^ Despite this, a detailed understanding is still lacking as the small length scales involved and the transient nature of the atomic-scale interactions prevent systematic experimental characterization.

Atomic-scale features also provide additional light localization which becomes particularly strong in “plasmonic” nanogaps, which are crevices between coinage metal nanostructures.^10,19,20^ These facilitate the optical isolation of any molecules nearby and provide an additional ∼10–100× optical field enhancement, benefiting optical interrogation techniques such as SERS.^3,19,21,22^ Such atomic-scale features, termed *picocavities*, tend to produce new sets of Raman lines because their small feature size generates strong field gradients across a molecule, probing normally Raman inactive modes as well as the active modes.^21,23^ In addition, the undercoordinated protruding metal atom can interact with the molecule, resulting in a shift in vibrational peaks.^3,24^ The wide range of possible interactions between the metallic protrusions and the analyte molecule makes repetitions rare, and signals are difficult to interpret without large amounts of data.

Here we present a unique analysis pipeline for SERS data that is inspired by image processing and machine learning techniques. Our workflow allows rapid and reliable processing of large SERS data sets, segmenting the transient features from the steady state. Utilizing a one-dimensional convolutional autoencoder (CAE), we can reliably reconstruct and subtract steady-state “nanocavity” spectra. This is followed by iterative thresholded detection on the residual spectra to identify the picocavity peaks. These isolated single molecule spectra are then clustered by similarity to identify recurring interactions and used to resolve, on an atomic level, where the adatoms are formed with respect to the probed molecule. To test the robustness of this approach, different sample types are explored, including commercial Au nanoparticles (NPs), in-house synthesized Au NPs, and Au samples with monolayer Pd atoms on either the nanoparticle or substrate, suppressing the formation of picocavities.

A nanoparticle-on-mirror (NPoM) geometry is chosen for its strong optical field enhancement and high reproducibility, while allowing for a large number of structures to be probed.^25^ A self-assembled monolayer (SAM) of the BPT (biphenyl-4-thiol) molecule is selected as a spacer for its high Raman cross section^26^ and rigid structure with few conformational isomers, providing stable SERS spectra eliminating confounding factors (Figure 1a). Note that this analysis can however be applied to a wide range of molecular structures and will be particularly valuable in studying molecular catalysis^27^ and molecular electronics.^28^

**Figure 1:**
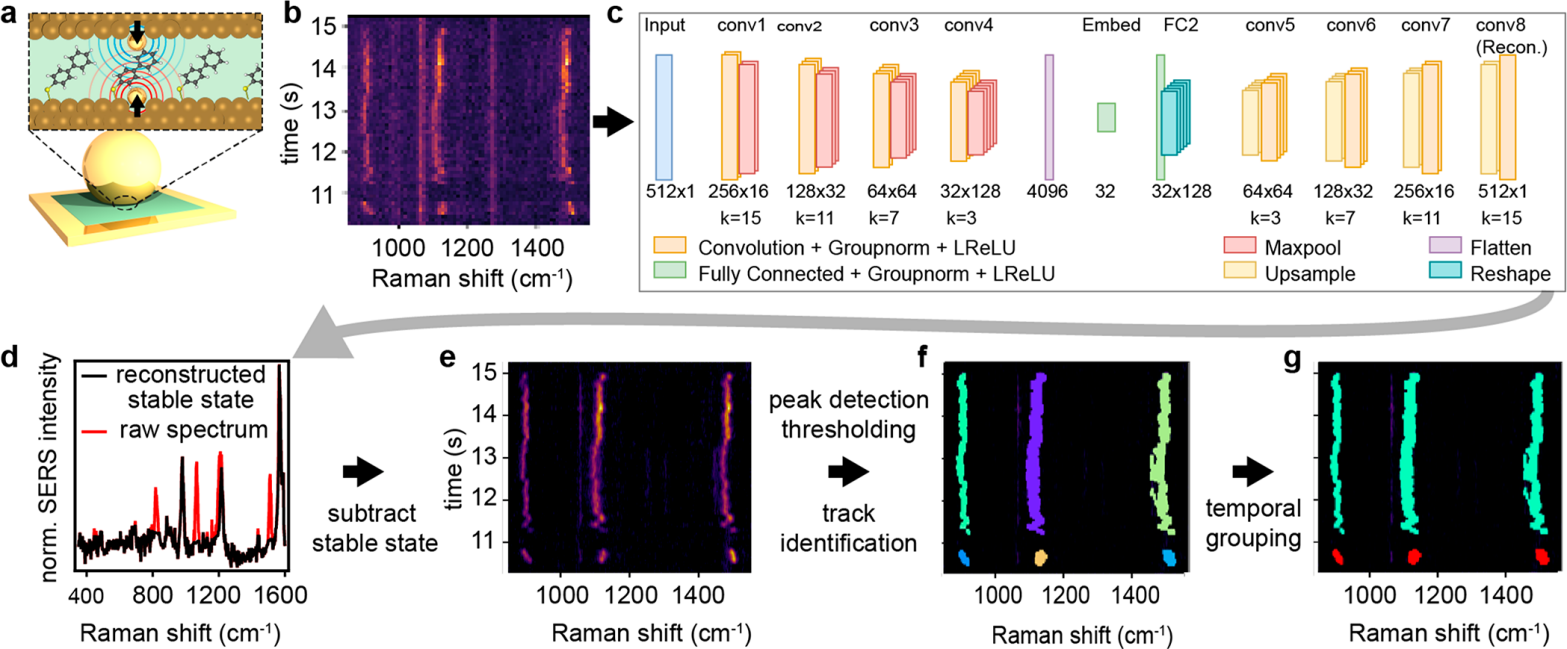
Isolating single molecule spectra. (a) Scheme depicting a nanoparticle-on-mirror (NPoM) geometry with atomic-scale protrusions in the nanogap. The 80 nm Au nanoparticle sandwiches the BPT molecular monolayer above the Au mirror. Inset above shows a 1.1 nm thick gap with molecules and single Au atoms being pulled out of facets. (b) Sequential SERS spectra at successive times from a NPoM showing both stable nanocavity lines and transient picocavity lines. (c) Block diagram of the convolutional autoencoder (CAE) architecture trained to reconstruct stable state spectra. (d) Reconstructed spectra (black) vs raw data (red). (e) Isolated picocavity spectra. (f) Contiguous picocavity peaks termed *tracks* are identified. (g) Tracks belonging to the same time series of picocavity spectra are formed into *events*, labeled by color.

To study picocavity events, a large SERS spectra database of NPoM is collected on a custom-built Raman microscope.^29^ Using 633 nm excitation, 1415000 spectra are collected over 1415 scans using three different laser powers: 447, 564, and 709 μW. These spectra are collected using 35 ms integration times, here termed one time step. During irradiation, picocavity signals appear as stochastic transient events (Figure 1b) and can last for up to several seconds. From these scans, 416 contain such picocavity events while 999 scans consist only of persistent nanocavity spectra. Reliable decomposition is imperative to build a representative data set; otherwise there is a risk of retaining nanocavity peaks or missing transient peaks overlapping in energy with nanocavity peaks, which could lead to the potential misclassification of a particular picocavity type. As noted above, BPT produces stable SERS signals, which result in nanocavity peaks appearing at consistent wavenumbers with pseudostable peak ratios and background intensities. These three features are termed the *stable state* of the BPT SERS spectra. Leveraging this consistency, a neural network was trained to readily adapt to any variances in the stable state to achieve robust isolation of the transient peaks and their corresponding event characteristics for further analysis.

The neural network architecture developed for this task is a one-dimensional convolutional autoencoder (CAE, Figure 1c), which is an unsupervised, shift-equivariant^30^ model that (once trained) is capable of extracting complex, salient features of an unlabeled data set by generating a method of nonlinear data compression. It is worth noting that this nonlinearity allows a more representative characterization of the steady state than would be afforded by calculating a simple time-averaged spectrum. The CAE was trained and validated on its ability to reconstruct the stable state of spectra belonging to scans containing only persistent nanocavity signals. During the test time, termed inference, scans that are found to contain picocavity events are processed by the CAE, which is only capable of reconstructing the stable state portions of their spectra (Figure 1d). It is noted that the labels used to partition the BPT database into the three described data sets are not used to train the CAE; thus, by comparing input and reconstructed signals, the CAE trains in an unsupervised manner, requiring no prerequisite knowledge about the shape of picocavity spectra. Once trained, each scan of reconstructed spectra, alongside their corresponding input spectra, is smoothed using a Savitzky–Golay filter (with a window length of 7 pixels and fit to a second-order polynomial). Each smoothed reconstruction *R*_λ,*t*_ is subtracted from its corresponding input spectrum *l*_λ,*t*_ with the inclusion of an offset parameter φ, which is equal to 5% of the standard deviation of the input scan, to give
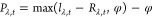
The purpose of the offset parameter is to only allow for signals associated with transient peaks to remain as the residual intensities after the subtraction of the input spectra while also minimizing the number of false detections due to noise. For example, a high signal-to-noise (SNR) scan would have a small offset and a correspondingly lower picocavity detection threshold that increases the sensitivity to weaker picocavity signals. The values of the Savitzky–Golay filter and offset parameters are selected through an empirical test on a representative subset of the BPT data. *P*_λ,*t*_ is termed the *picocavity scan* (Figure 1e), which forms the basis for further processing stages. Once trained, the CAE can rapidly process large amounts of data (see Figure S1).

For all spectra, a background of 300 counts (CCD dark current) is subtracted, and the data are normalized between [0, 1], as varying intensities would intrinsically bias how a neural network trains. Spectra are cropped to the wavelength range of interest (268 cm^–1^, 1611 cm^–1^) and interpolated using a cubic spline to produce 512 equal-width bins per spectrum with a resolution of 2.6 cm^–1^. Interpolating the data in this way aligns the attributes (the wavenumbers) of all spectra within the data set and alleviates a parameter bias incurred from a nonuniform resolution wavenumber space.^31^ Lastly, during each training epoch, random uniform noise (proportional to the square root of the signal; designed to mimic the noise produced by the dispersive detector) is exclusively added to the training data set in order to increase sample variance. This technique is a form of data augmentation, in which additional training samples are synthesized by applying application-specific transformations or (in this case) noise to produce a more robust network.^32^ This additional noise is only used for training and is not applied during the peak detection process and thus cannot contribute to the false identification of a transient peak.

The CAE contains nine hidden layers including four encoding convolutional blocks in the encoder followed by a 32-unit fully connected (FC) embedding, which serves as an input for the decoder. The decoder mirrors the encoder architecture with one FC layer whose output is reshaped to fit the next convolutional layer and four convolutional blocks that upscale the data to reconstruct the input spectra. The outputs of each layer are normalized using group normalization,^33^ followed by a Leaky ReLU^34,35^ activation function with a slope coefficient, α = 0.3. Leaky ReLU was chosen over standard ReLU to prevent the “dying ReLU” problem.^36^ Maxpooling, with a stride and kernel size of two, is used as the final layer within each convolutional block of the encoder to downsample each feature map, and upscaling with a factor of 2 is used as the first layer within each convolutional block of the decoder. The model depth and size for each layer were determined through a grid search optimization, minimizing the mean-square-error (MSE) loss. Figure 1c shows a block diagram of the CAE architecture.

The model is trained for 2500 epochs, chosen to jointly minimize overfitting and training times, using a static learning rate of 0.001 and a batch size of 500 spectra. The training data set consists of 749 scans that only contain persistent nanocavity signals, while the validation data set consists of the remaining 250 stable scans. The testing data set contains all 416 scans that contain picocavity events. The MSE loss is used with the Adam optimization algorithm^37^ (using parameters β_1_ = 0.9, β_2_ = 0.999, and ϵ = 10^–7^) to adjust model parameters during training. Each layer is regularized using L2 weight decay with a regularization factor, γ = 0.1. Clipnorm^38^ is used to clip the calculated gradients to the maximum L2-norm value of each update step to avoid the problem of diverging gradients.

Once *P*_λ,*t*_ is generated, the pixel locations of the most intense transient peaks are isolated by a 98th percentile pixel intensities threshold. To detect lower intensity peaks, a 96th percentile threshold is applied with the addition of a Boolean mask to allow only pixels that share rows or columns with previous detections. These two stages capture the majority of pixels containing transient peaks in most scans; however, scans with a lower SNR are found to contain small gaps in otherwise complete sequential transient peaks in a time series, called *tracks* (Figure 1f). To solve this, two probability density functions are estimated by counting the number of peaks detected along each axis. Additional detections are made, in a similar fashion to the previous methods, by fitting a scaling percentile range to the probability density functions between the 90th and 96th percentiles, where the lower percentile bound is applied to the highest count, and vice versa. These percentile functions are applied along their respective axes, and an intersection of their detections forms the final coordinate set of transient peaks in pixel space, combined with the previously detected positions. A basic empirical study is performed on a representative subset of the testing data, showing that the percentile values used provide the best results.

As each detection stage utilizes percentiles to detect peaks, intense noise can also be detected, which could connect two or more otherwise distinct tracks. To prevent the accumulation of noise, morphological opening is applied to the output scan at each detection stage using a (3 × 3) rectangular structuring element. Initial tracks are demarcated by the final detected pixels that are 8-connected (meaning that they share an edge or a vertex) in each scan. Identifying all tracks within a single picocavity event is crucial; therefore, an algorithm was created to merge tracks that had become disconnected due to either algorithmic error or intensity variations caused by physical effects such as thermal fluctuations from a bistable picocavity.^11,22,24^ This algorithm, here termed the *zipper*, examines tracks near one another by utilizing a sliding window with dimensions (3 × 10) pixels fixed to the mean wavenumber position of each track segment and initially placed at the earliest time step shared between both tracks. The sliding window moves through later time steps with a stride of one and stores the mean wavenumber separation between the centroids of each transient peak. Once all valid window positions have been examined, the global mean separation is calculated between the two tracks, and they are merged if their separation falls below a tolerance value of 5 pixels, which translates to approximately 13 cm^–1^ specified to encapsulate the full range of expected peak drifts.

Once the zipper algorithm is complete, the resulting tracks extracted from the SERS scans make up the data set for subsequent analysis stages, as in Figure 1f. As SERS signals retrieved from picocavities typically contain multiple peaks that appear and disappear simultaneously, the next stage is to match tracks belonging to the same picocavity event, herein termed *events*. Because of errors in peak detections discussed above, any particular track may be incomplete. A pairwise comparison is made between tracks, in which the ratio of the number of shared time steps to the duration of the longest track is calculated. If the ratio of any pair exceeds a threshold of 0.7 (empirically determined using a representative subset of BPT data), then those tracks are assigned to the same event; see Figure 1g for an example.

Each event represents one instance of physical picocavity formation. With enough data, reoccurring events can be clustered based on similarity to represent a narrowed set of event types corresponding to a localized adatom–molecule interaction, here termed a *configuration*. In order to form clusters, the normalized mean picocavity spectrum of each event is first compared with others using the Wasserstein distance metric, which calculates the amount of work needed to transform from one probability distribution to another. This forms an (*N* × *N*) distance matrix, where *N* is in the total number of events. Spectral clustering^41^ was chosen as the clustering method, which requires a similarity matrix; hence, the radial basis function kernel is applied to the distance matrix for this conversion. Spectral clustering requires the number of clusters to be prespecified. However, because the number of unique configurations is unknown, the number of clusters with the highest mean silhouette coefficient^42^ was selected from a range of 2–30. The silhouette scores of the events for the top four clusters are depicted in Figure 2b showing the range of cluster separations.

**Figure 2:**
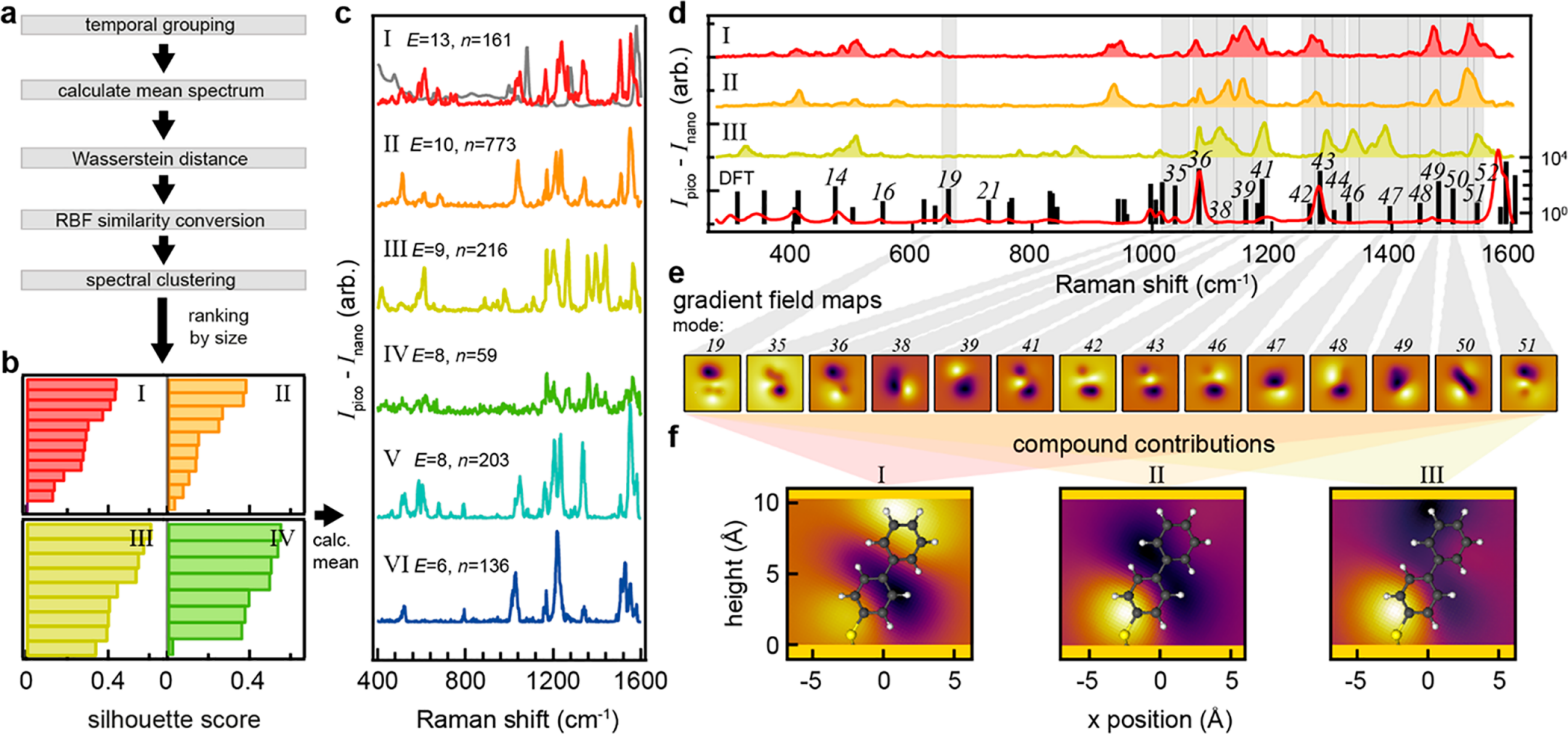
(a) Picocavity event grouping pipeline. (b) Silhouette scores for events in the four largest clusters (I–IV) where 1 means identical and 0 means no correlation. (c) Grouped picocavity event spectra scaled by their silhouette score representing distinct molecule–picocavity interactions termed *configurations*, presented in descending order of frequency labeled I–VI, with the global nanocavity spectrum shown in gray, events labelled as *E*, number of spectra as *n*. (d) Peak range assignment for the three most common configurations based on stable state DFT modeling of BPT. (e) Mapped gradient field Raman, yielding a local response map for each of the assigned modes using a method adapted from ref (39). (f) Compounded local response maps for configurations I–III, plotted against a geometry-optimized BPT-Au molecule, depicted at a 29° angle from the surface normal.^40^ (Note: both the local response/compound maps and BPT molecule are depicted normal to the molecule’s phenyl rings to better visualize distributions; in reality a more planar geometry with respect to the surface is expected.)

Occasionally rapid on–off switching of a picocavity interaction is observed with near identical spectra, which causes these to be counted as multiple events if there is sufficient time separation between each occurrence. This is more prevalent in Pd-functionalized NPoM geometries (discussed below) and can result in a bias toward switching-type configurations at the clustering stage. To account for this, events that are clustered together and originate from the same scan are merged if they are within 100 time steps of each other (Figure S3). Then, the original clusters are dissolved, and the spectral clustering method is repeated using refined events. Lastly, individual members within each cluster are discarded if their silhouette sample scores are negative. From this evaluation, six clusters achieved the best silhouette coefficient score. The representative spectra for each configuration are shown in Figure 2c.

The ranked configuration spectra each show a unique combination of SERS peaks that are absent or only weakly present in the nanocavity spectrum shown in gray (Figure 2c). The Raman spectrum of a BPT molecule bound to a single gold atom was simulated using the commercial density functional theory (DFT) package Gaussian 09W, employing the B3LYP hybrid functional with the (Def2TZVP) basis set and D3 dispersion correction with Becke–Johnson damping.^43^ A good agreement is found with the stable nanocavity spectrum calculated from all combined reconstructed stable states, allowing tentative assignments to be made for each vibrational mode (Figure S3). However, in experiments, a broadened range of possible picocavity peak positions is observed. Considering there are only a limited number of vibrations available in the rigid BPT molecule, the range for each vibrational mode can be estimated based on the drift in peak position observed experimentally over the duration of individual events (example shown in Figure S4).

By adapting the method for calculating the Raman response of a molecule in an inhomogeneous field, developed by Aizpurua et al.,^39^ a simplified analytical expression for the picocavity field gradient^44^ can be swept along the molecule (Figure S5). This provides a local gradient field response map and is repeated for each vibrational mode. (Figure 2e). This visualizes how vibrational modes respond differently to gradient fields across the molecule. Using this, a compound local response map is generated by averaging together the local gradient field maps for each identified vibrational mode multiplied by the corresponding peak area. To eliminate any systematic bias, the near-field maps are normalized by the average of all gradient field maps combined (Figure 2f).
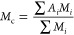
where *M*_c_ is the compounded gradient-field Raman map, *A*_*i*_ the peak area corresponding to vibration *i*, and *M*_*i*_ the gradient Raman map for vibration *i*. The resulting near-field maps provide an insight into the location where the field gradient originates from around the molecule and can be used to tentatively assign the position of the atomic-scale feature giving rise to the highly localized field (e.g., adatom). For configuration I (the largest cluster: 13 events, 161 spectra) the near-field map suggests picocavities arise near the upper nanoparticle (NP type) whereas all five other configurations (with a combined 41 events made up of 1387 spectra) indicate picocavities form near the substrate (Figure S6). These findings show that the adatoms giving rise to the picocavity spectra are most likely to originate from the substrate (10:90%; NP vs substrate), which agrees with previous work where a distinguishable Raman marker (cyanide group) was included in the molecule to determine the “NP” vs “substrate” picocavity ratio (15:85%).^25^ However, 24% of events are classed as coming from the NP showing that substrate events contain on average more spectra (34 vs 12), showing that therefore these events persist for longer.

To verify the validity of these findings and test the robustness of the approach, a second database was prepared using a second bespoke Raman microscope with a higher spectral resolution (resulting in narrower peaks), using longer integration times (0.20 s), and in-house synthesized AuNPs instead. To further validate the assignment of NP vs substrate picocavities, two additional NPoM varieties were prepared, where a monolayer of palladium is grown, which is found to suppress the formation of picocavities on either the AuNP or the substrate,^45^ in line with predicted adatom formation energy costs.^46^ These sample varieties are here labeled by the NP and substrate metal M as M_NP_–M_substrate_ as Au–Au, Au–Pd/Au, and Au@Pd–Au (Figure 3a).

**Figure 3:**
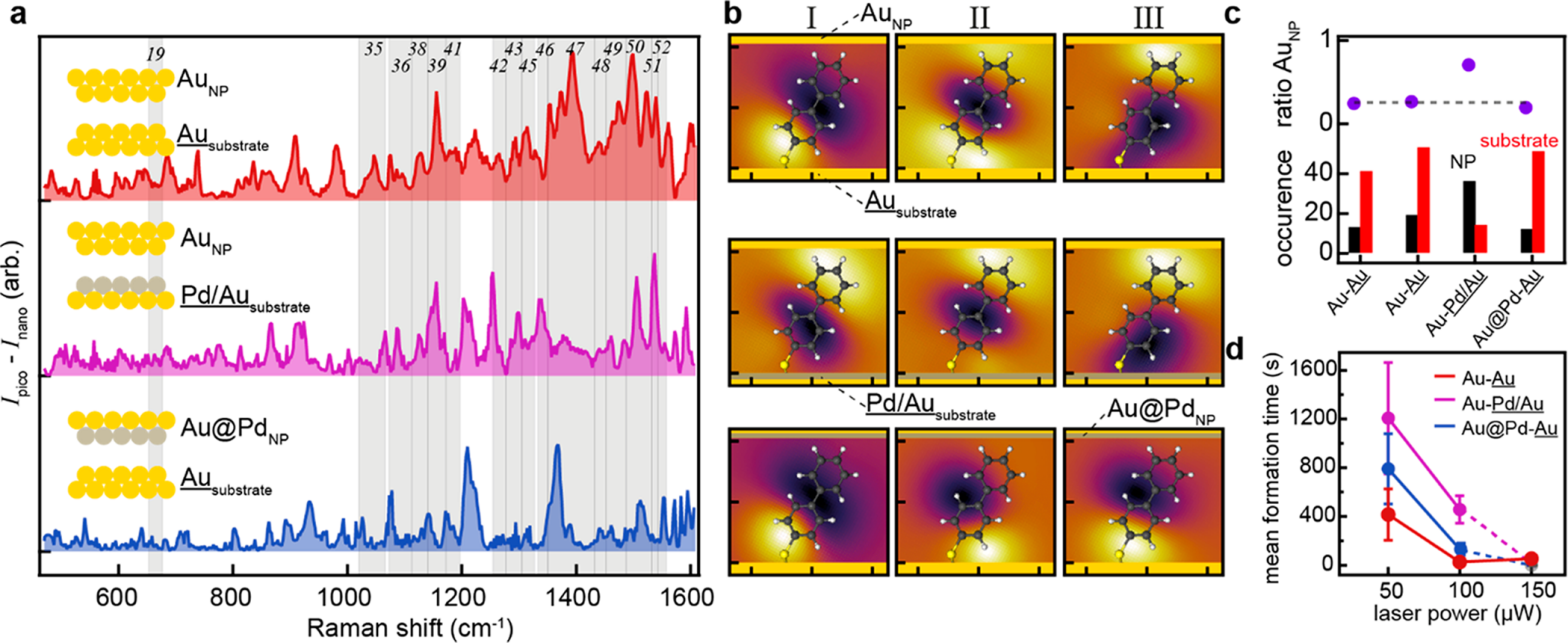
(a) The most frequent configuration for each of the three additional NPoM varieties made using in-house synthesized AuNPs and depositions of a monolayer Pd atoms on either the nanoparticle or the substrate. (B) Top three near-field maps for each configuration. (C) Occurrence and ratios of events for each sample variety comparing adatom formation on the NP vs substrate. (D) Mean formation times for each configuration as a function of laser power.

The new database contains 1833500 spectra over 3667 scans (500 spectra/scan), of which 3479 scans contain picocavities and 188 contain only nanocavity signals (note: due to the longer duration of each scan, fewer consist of only nanocavity spectra). The nanocavity data are partitioned into 144 scans for the training data set and 44 for the validation data set. Because of the limited number of stable nanocavity spectra split between the three new NPoM varieties, the existing pretrained CAE parameters are fine-tuned on the nanocavity data within the new training data set. Despite a limited number of stable nanocavity spectra being used, the algorithm was readily adapted to the spectral properties of the new data. After training, each NPoM variety is assessed separately as generating 6, 6, and 10 event types after independent clustering for Au–Au, Au–Pd/Au, and Au@Pd–Au samples, respectively. The spectrum of the most common configuration for each NPoM variety is shown in Figure 3a, with Figure 3b showing the near-field maps of the three most common events for each (all other near-field maps are shown in Figures S7–S9). The homemade Au–Au NPoM shows configurations I, II, IV, and V indicative of substrate picocavities (total 53 events over 4511 spectra) with the NP picocavities now split over configurations III and VI (19 events, 976 spectra). This shows 15:85% of picocavity spectra come from the NP with 26:74% of events classed as NP, in close agreement with the previous observations and literature.^25^

Conversely, when a Pd monolayer is introduced on the substrate, all but one configuration (VI) show picocavities forming from the NP (Figures 3b and S8), with one containing a mixture of NP and substrate contributions (IV). This results in 90:10% of picocavity spectra now originating from the NP and 88:12% of events (excluding mixed configuration IV). In addition, in contrast to the previous observations, slightly more spectra are observed on average per event for those coming from the NP vs from the substrate (92 vs 72). For the sample type where the NP is coated in a monolayer of Pd, 10 clusters are found. Of these, seven configurations show substrate events and three NP events (Figures 3b and S9) with only 19% of events now coming from the NP (an ∼5% drop with respect to Au–Au samples. The average spectra per event for the NP types greatly increased, with on average 47 spectra per event for the NPs vs 20 for the substrate.

Overall by this spatially-resolved comparison of the events from each of the samples, the strong effects from coating either surface with a Pd monolayer become clearly visible. The Pd coating suppresses the formation of adatoms on the newly functionalized surface (Figure 3c). To confirm this suppression, the mean picocavity formation times for all three variants are compared at different laser powers. This shows a similar trend, with Pd-coated substrates having the strongest effect on the formation rate, i.e., longer mean formation time (Figure 3d), agreeing well with predictions in the literature.^46^

To conclude, we introduced a robust method to extract salient features from SERS spectra and used this to isolate and cluster a large number of picocavity spectra. We also show that by adapting an existing inhomogeneous field Raman mapping method, a tentative position for adatoms can be extracted. Using this method, we find that the formation rate, location, and lifetime of picocavities can be influenced by functionalizing either the substrate or the NPs with a monolayer of Pd atoms. This now provides a unique insight into the formation behavior and the coordination geometries of adatoms in metal surfaces. This deep learning technique will translate to many other analyte molecules as long as steady-state spectra can be acquired for training purposes. We thus believe the data analysis pipeline introduced here offers a powerful tool to assist in the rational design of heterogeneous catalysts as well as for more general analysis of spectra across physics and chemistry. We aim to make our code publicly available on GitHub.

## Supporting Information Available

Comparison ALS vs CAE; example on/off switching of picocavities; peak assignment; example picocavity peak shift range; analytical approximation for picocavity field; gradient field maps I–VI (Au–Au) for commercial NPs; gradient field maps I–VI (Au–Au) for homemade NPs; gradient field maps I–VI (Au–Pd@Au) for homemade NPs; gradient field maps I–X (Pd@Au–Au) for homemade NPs (PDF)

## References

[ref1] Yang X.-F.; Wang A.; Qiao B.; Li J.; Liu J.; Zhang T. Single-Atom Catalysts: A New Frontier in Heterogeneous Catalysis. Acc. Chem. Res. 2013, 46 (8), 1740–1748. 10.1021/ar300361m. 23815772

[ref2] Qin S.; Will J.; Kim H.; Denisov N.; Carl S.; Spiecker E.; Schmuki P. Single Atoms in Photocatalysis: Low Loading Is Good Enough. ACS Energy Lett. 2023, 8 (2), 1209–1214. 10.1021/acsenergylett.2c02801.

[ref3] Griffiths J.; Földes T.; de Nijs B.; Chikkaraddy R.; Wright D.; Deacon W. M.; Berta D.; Readman C.; Grys D.-B.; Rosta E.; Baumberg J. J. Resolving Sub-Angstrom Ambient Motion through Reconstruction from Vibrational Spectra. Nat. Commun. 2021, 12 (1), 6759 10.1038/s41467-021-26898-1. 34799553 PMC8604935

[ref4] Li X.; Yang X.; Zhang J.; Huang Y.; Liu B. In Situ/Operando Techniques for Characterization of Single-Atom Catalysts. ACS Catal. 2019, 9 (3), 2521–2531. 10.1021/acscatal.8b04937.

[ref5] An H.; Wu L.; Mandemaker L. D. B.; Yang S.; Ruiter J.; Wijten J. H. J.; Janssens J. C. L.; Hartman T.; Stam W.; Weckhuysen B. M. Sub-Second Time-Resolved Surface-Enhanced Raman Spectroscopy Reveals Dynamic CO Intermediates during Electrochemical CO_2_ Reduction on Copper. Angew. Chem., Int. Ed. 2021, 60 (30), 16576–16584. 10.1002/anie.202104114. PMC8362134 33852177

[ref6] Liu J.; Osadchy M.; Ashton L.; Foster M.; Solomon C. J.; Gibson S. J. Deep Convolutional Neural Networks for Raman Spectrum Recognition: A Unified Solution. Analyst 2017, 142 (21), 4067–4074. 10.1039/C7AN01371J. 28993828

[ref7] Angulo A.; Yang L.; Aydil E. S.; Modestino M. A. Machine Learning Enhanced Spectroscopic Analysis: Towards Autonomous Chemical Mixture Characterization for Rapid Process Optimization. Digit. Discovery 2022, 1 (1), 35–44. 10.1039/D1DD00027F.

[ref8] Liu J.; Gibson S. J.; Mills J.; Osadchy M. Dynamic Spectrum Matching with One-Shot Learning. Chemom. Intell. Lab. Syst. 2019, 184, 175–181. 10.1016/j.chemolab.2018.12.005.

[ref9] Xie W.; Walkenfort B.; Schlücker S. Label-Free SERS Monitoring of Chemical Reactions Catalyzed by Small Gold Nanoparticles Using 3D Plasmonic Superstructures. J. Am. Chem. Soc. 2013, 135 (5), 1657–1660. 10.1021/ja309074a. 23186150

[ref10] Benz F.; Schmidt M. K.; Dreismann A.; Chikkaraddy R.; Zhang Y.; Demetriadou A.; Carnegie C.; Ohadi H.; de Nijs B.; Esteban R.; Aizpurua J.; Baumberg J. J. Single-Molecule Optomechanics in “Picocavities”. Science 2016, 354 (6313), 726–729. 10.1126/science.aah5243. 27846600

[ref11] Huang J.; Grys D.-B.; Griffiths J.; de Nijs B.; Kamp M.; Lin Q.; Baumberg J. J. Tracking Interfacial Single-Molecule PH and Binding Dynamics via Vibrational Spectroscopy. Sci. Adv. 2021, 7 (23), eabg1790 10.1126/sciadv.abg1790. 34088670 PMC8177700

[ref12] Wu T.; Yan W.; Lalanne P. Bright Plasmons with Cubic Nanometer Mode Volumes through Mode Hybridization. ACS Photonics 2021, 8 (1), 307–314. 10.1021/acsphotonics.0c01569.

[ref13] Park W.-H.; Kim Z. H. Charge Transfer Enhancement in the SERS of a Single Molecule. Nano Lett. 2010, 10 (10), 4040–4048. 10.1021/nl102026p. 20857978

[ref14] Pfisterer J. H. K.; Liang Y.; Schneider O.; Bandarenka A. S. Direct Instrumental Identification of Catalytically Active Surface Sites. Nature 2017, 549 (7670), 74–77. 10.1038/nature23661. 28880284

[ref15] Back S.; Yeom M. S.; Jung Y. Active Sites of Au and Ag Nanoparticle Catalysts for CO_2_ Electroreduction to CO. ACS Catal. 2015, 5 (9), 5089–5096. 10.1021/acscatal.5b00462.

[ref16] Thompson D.; Liao J.; Nolan M.; Quinn A. J.; Nijhuis C. A.; O’Dwyer C.; Nirmalraj P. N.; Schönenberger C.; Calame M. Formation Mechanism of Metal–Molecule–Metal Junctions: Molecule-Assisted Migration on Metal Defects. J. Phys. Chem. C 2015, 119 (33), 19438–19451. 10.1021/acs.jpcc.5b04383.

[ref17] Raffone F.; Risplendi F.; Cicero G. A New Theoretical Insight Into ZnO NWs Memristive Behavior. Nano Lett. 2016, 16 (4), 2543–2547. 10.1021/acs.nanolett.6b00085. 26928559

[ref18] Lyu S.; Zhang Y.; Zhang Y.; Chang K.; Zheng G.; Wang L. Picocavity-Controlled Subnanometer-Resolved Single-Molecule Fluorescence Imaging and Mollow Triplets. J. Phys. Chem. C 2022, 126 (27), 11129–11137. 10.1021/acs.jpcc.2c00531.

[ref19] Urbieta M.; Barbry M.; Zhang Y.; Koval P.; Sánchez-Portal D.; Zabala N.; Aizpurua J. Atomic-Scale Lightning Rod Effect in Plasmonic Picocavities: A Classical View to a Quantum Effect. ACS Nano 2018, 12 (1), 585–595. 10.1021/acsnano.7b07401. 29298379

[ref20] Richard-Lacroix M.; Deckert V. Direct Molecular-Level near-Field Plasmon and Temperature Assessment in a Single Plasmonic Hotspot. Light Sci. Appl. 2020, 9 (1), 35 10.1038/s41377-020-0260-9. 32194949 PMC7061098

[ref21] Benz F.; Schmidt M. K.; Dreismann A.; Chikkaraddy R.; Zhang Y.; Demetriadou A.; Carnegie C.; Ohadi H.; de Nijs B.; Esteban R.; Aizpurua J.; Baumberg J. J. Single-Molecule Optomechanics in “Picocavities. Science 2016, 354 (6313), 726–729. 10.1126/science.aah5243. 27846600

[ref22] Griffiths J.; de Nijs B.; Chikkaraddy R.; Baumberg J. J. Locating Single-Atom Optical Picocavities Using Wavelength-Multiplexed Raman Scattering. ACS Photonics 2021, 8 (10), 2868–2875. 10.1021/acsphotonics.1c01100. 34692898 PMC8532146

[ref23] Shin H.-H.; Yeon G. J.; Choi H.-K.; Park S.-M.; Lee K. S.; Kim Z. H. Frequency-Domain Proof of the Existence of Atomic-Scale SERS Hot-Spots. Nano Lett. 2018, 18 (1), 262–271. 10.1021/acs.nanolett.7b04052. 29206468

[ref24] Lin Q.; Hu S.; Földes T.; Huang J.; Wright D.; Griffiths J.; Elliott E.; de Nijs B.; Rosta E.; Baumberg J. J. Optical Suppression of Energy Barriers in Single Molecule-Metal Binding. Sci. Adv. 2022, 8 (25), eabp9285 10.1126/sciadv.abp9285. 35749500 PMC9232110

[ref25] Carnegie C.; Griffiths J.; de Nijs B.; Readman C.; Chikkaraddy R.; Deacon W. M.; Zhang Y.; Szabó I.; Rosta E.; Aizpurua J.; Baumberg J. J. Room-Temperature Optical Picocavities below 1 Nm3 Accessing Single-Atom Geometries. J. Phys. Chem. Lett. 2018, 9 (24), 7146–7151. 10.1021/acs.jpclett.8b03466. 30525662

[ref26] Anderson A. G. Jr.; Steckler B. M. Azulene. VIII. A Study of the Visible Absorption Spectra and Dipole Moments of Some 1- and 1,3-Substituted Azulenes1,2. J. Am. Chem. Soc. 1959, 81 (18), 4941–4946. 10.1021/ja01527a046.

[ref27] Wright D.; Lin Q.; Berta D.; Földes T.; Wagner A.; Griffiths J.; Readman C.; Rosta E.; Reisner E.; Baumberg J. Research Mechanistic Study of an Immobilised Molecular Electrocatalyst by in-Situ Gap Plasmon Assisted Spectro-Electrochemistry. Nat. Catal. 2021, 4, 157–169. 10.1038/s41929-020-00566-x.

[ref28] Komoto Y.; Fujii S.; Iwane M.; Kiguchi M. Single-Molecule Junctions for Molecular Electronics. J. Mater. Chem. C 2016, 4 (38), 8842–8858. 10.1039/C6TC03268K.

[ref29] Benz F.; Chikkaraddy R.; Salmon A.; Ohadi H.; de Nijs B.; Mertens J.; Carnegie C.; Bowman R. W.; Baumberg J. J. SERS of Individual Nanoparticles on a Mirror: Size Does Matter, but so Does Shape. J. Phys. Chem. Lett. 2016, 7 (12), 2264–2269. 10.1021/acs.jpclett.6b00986. 27223478 PMC4916483

[ref30] Mouton C.; Myburgh J. C.; Davel M. H. Stride and Translation Invariance in CNNs. Southern African Conference for Artificial Intelligence Research, 2020; pp 267–281.

[ref31] Basri R.; Galun M.; Geifman A.; Jacobs D.; Kasten Y.; Kritchman S. Frequency bias in neural networks for input of non-uniform density. International Conference on Machine Learning (PMLR) 2020, 119, 685–694.

[ref32] An G. The Effects of Adding Noise During Backpropagation Training on a Generalization Performance. Neural Comput 1996, 8 (3), 643–674. 10.1162/neco.1996.8.3.643.

[ref33] Wu Y.; He K. Group Normalization. Proc. of the Eur. conf. on computer vision (ECCV). 2018, 11217, 3–19. 10.1007/978-3-030-01261-8_1.

[ref34] Maas A. L.; Hannun A. Y.; Ng A. Y. Rectifier Nonlinearities Improve Neural Network Acoustic Models. Proc. Icml. 2013, 30 (1), 3.

[ref35] Xu B.; Wang N.; Chen T.; Li M. Empirical Evaluation of Rectified Activations in Convolutional Network. arXiv: 1505.00853, 2015.

[ref36] Lu L.; Shin Y.; Su Y.; Karniadakis G. E. Dying ReLU and Initialization: Theory and Numerical Examples. Commun. Comput. Phys. 2020, 28 (5), 1671–1706. 10.4208/cicp.OA-2020-0165.

[ref37] Kingma D. P.; Ba J. Adam: A Method for Stochastic Optimization. International conference on learning representations (ICLR) 2015, 5, 6.

[ref38] Pascanu R.; Mikolov T.; Bengio Y. On the Difficulty of Training Recurrent Neural Networks. International Conference on Machine Learning 2013, 28 (3), 1310–1318.

[ref39] Zhang Y.; Dong Z.-C.; Aizpurua J. Theoretical Treatment of Single-Molecule Scanning Raman Picoscopy in Strongly Inhomogeneous near Fields. J. Raman Spectrosc. 2021, 52 (2), 296–309. 10.1002/jrs.5991.

[ref40] Turchanin A.; Käfer D.; El-Desawy M.; Wöll C.; Witte G.; Gölzhäuser A. Molecular Mechanisms of Electron-Induced Cross-Linking in Aromatic SAMs. Langmuir 2009, 25 (13), 7342–7352. 10.1021/la803538z. 19485375

[ref41] Ng A. Y.; Jordan M. I.; Weiss Y. On Spectral Clustering: Analysis and an Algorithm. Advances in neural information processing systems (NIPS’01). 2001; Vol. 14, pp 849—856.

[ref42] Rousseeuw P. J. Silhouettes: A Graphical Aid to the Interpretation and Validation of Cluster Analysis. J. Comput. Appl. Math. 1987, 20, 53–65. 10.1016/0377-0427(87)90125-7.

[ref43] Grimme S.; Ehrlich S.; Goerigk L. Effect of the Damping Function in Dispersion Corrected Density Functional Theory. J. Comput. Chem. 2011, 32 (7), 1456–1465. 10.1002/jcc.21759. 21370243

[ref44] Baumberg J. J. Picocavities: A Primer. Nano Lett. 2022, 22 (14), 5859–5865. 10.1021/acs.nanolett.2c01695. 35793541 PMC9335881

[ref45] Hu S.; Lin Q.; Goerlitzer E. S. A.; de Nijs B.; Silkin V. M.; Baumberg J. J. Alchemically-Glazed Plasmonics Using Atomic Layer Metals: controllably synergizing catalysis and plasmonics. Manuscript in preparation. 10.1038/s41467-025-58578-9 PMC11982554 40204697

[ref46] Xu L.; Papanikolaou K. G.; Lechner B. A. J.; Je L.; Somorjai G. A.; Salmeron M.; Mavrikakis M. Formation of Active Sites on Transition Metals through Reaction-Driven Migration of Surface Atoms. Science 2023, 380 (6640), 70–76. 10.1126/science.add0089. 37023183

