# Correction to “Mapping Atomic-Scale Metal–Molecule Interactions: Salient Feature Extraction through Autoencoding of Vibrational Spectroscopy Data”

**DOI:** 10.1021/acs.jpclett.3c02706

**Published:** 2023-10-26

**Authors:** Alex Poppe, Jack Griffiths, Shu Hu, Jeremy J. Baumberg, Margarita Osadchy, Stuart Gibson, Bart de Nijs

Because of a production error, the affiliations of two authors were transposed. Here are the correct affiliations for Bart de Nijs and Stuart Gibson:

**Bart de Nijs**

NanoPhotonics Centre, Cavendish Laboratory, University of Cambridge, Cambridge CB3 0HE, U.K. Email: bd355@cam.ac.uk

**Stuart Gibson**

School of Physics and Astronomy, University of Kent, Canterbury CT2 7NH, U.K. Email: s.j.gibson@kent.ac.uk

