# Supplementary material for: Mapping Atomic-Scale Metal–Molecule Interactions: Salient Feature Extraction through Autoencoding of Vibrational Spectroscopy Data
Source: J Phys Chem Lett. 2023 Aug 18;14(34):7603–10. doi: 10.1021/acs.jpclett.3c01483 (PMC10476190; doi:10.1021/acs.jpclett.3c01483)
Supplement: Supplementary file 1 — jz3c01483_si_001.pdf [file jz3c01483_si_001.pdf]

Mapping Atomic-Scale Metal-Molecule Interactions; Salient Feature Extraction Through Autoencoding of Vibrational Spectroscopy Data

Supporting Information

Alex Poppe, Jack Griffiths, Shu Hu, Jeremy J. Baumberg, Margarita Osadchy, Stuart Gibson*, Bart de Nijs*

Keywords: *Autoencoder, Machine Learning, Feature Extraction, Surface-Enhanced Raman Spectroscopy, Picocavities*

Supplementary note 1: Briefly, an Olympus BX51 microscope was coupled to an Andor Shamrock 303i dispersive spectrometer, with a 600l/mm grating and an Andor Newton 970 BVF electron-multiplying CCD in conventional mode[1]. An in-house particle finding algorithm, using threshold detection on dark-field scattering images, was paired with an automated stage to collect spectra from 1,415 Particles[2]. Raman data was captured over a wavenumber range of -1606 cm^{-1} to 1611 cm^{-1}, with a non-linear resolution ranging between 2.5 cm^{-1} to 1.6 cm^{-1} across this spectrum, as a result of the dispersive detector. For the second database an Olympus BX51 microscope was coupled to a Horiba Triax 320 dispersive spectrometer with a 600l/mm grating and an Andor Newton 970 BVF electron-multiplying CCD in conventional mode. In both cases the laser is coupled in using a 10/90 beam splitter and the scattered laser light is blocked using two notch filters. In both cases spectra are collected using an Olympus 0.9 NA 100x darkfield objective (MPLFLN100xBD).

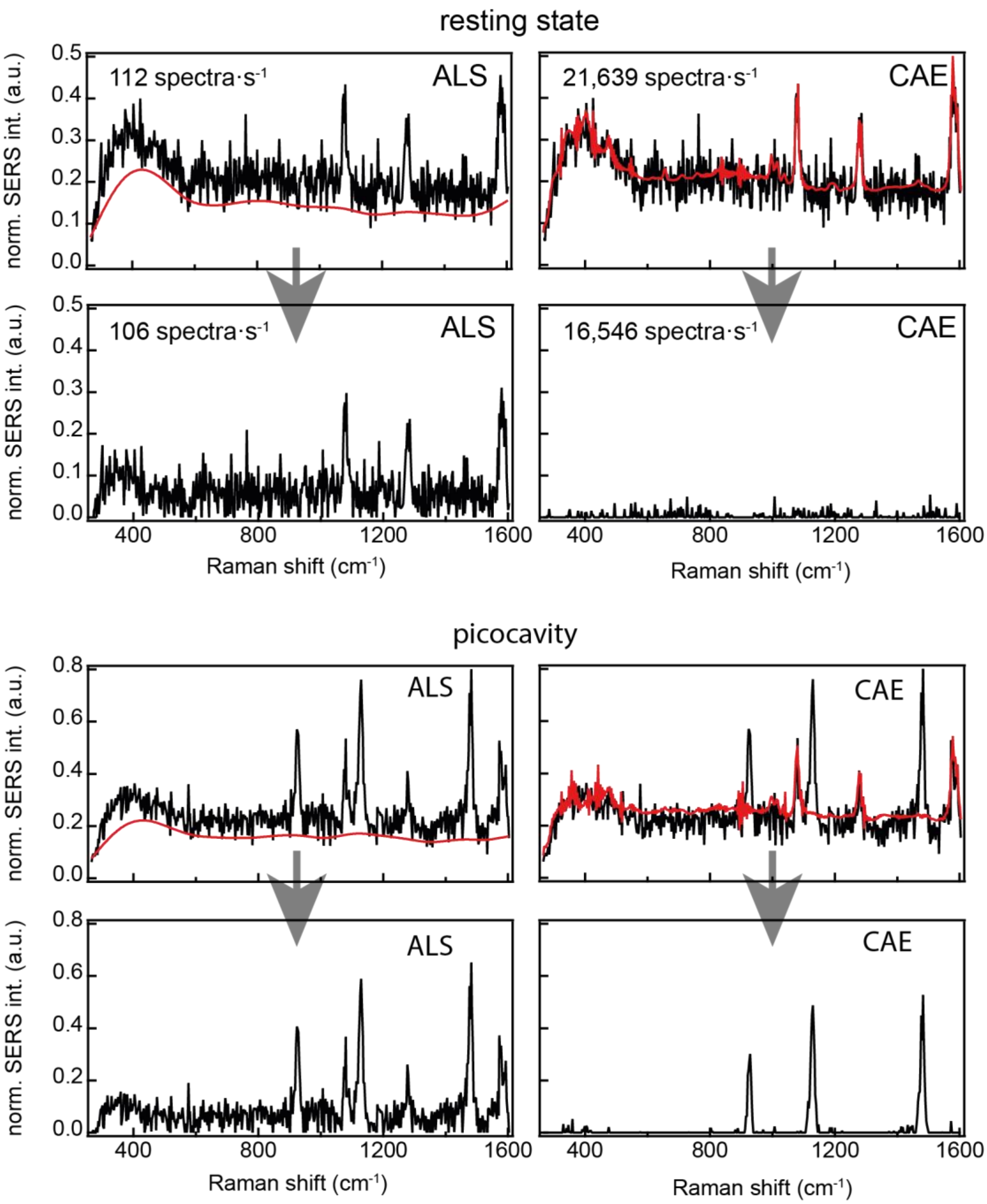

Figure S1: Comparison between common spectral processing algorithms: asymmetric Least Squares (ALS) as a background fitting and the convolutional autoencoder (CAE) introduced here. The CAE reconstructs the stable state but not the picocavity event allowing for isolation of an Event's spectral properties. Whilst the conventional background fitting method (ALS) only reconstructs part of the spectrum the process is nearly 200x slower. The CAE allows for >16,000 spectra to be processed per second, including subtraction, on a standard desktop computer.

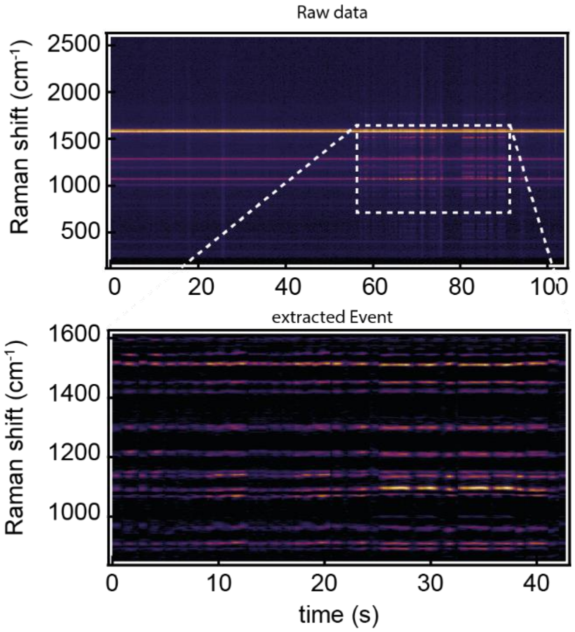

Figure S2: Example of on-off switching behaviour of picocavity signals on a Au-Pd@Au NPoM, shown as raw data (top) and from the extracted, isolated picocavity event (bottom).

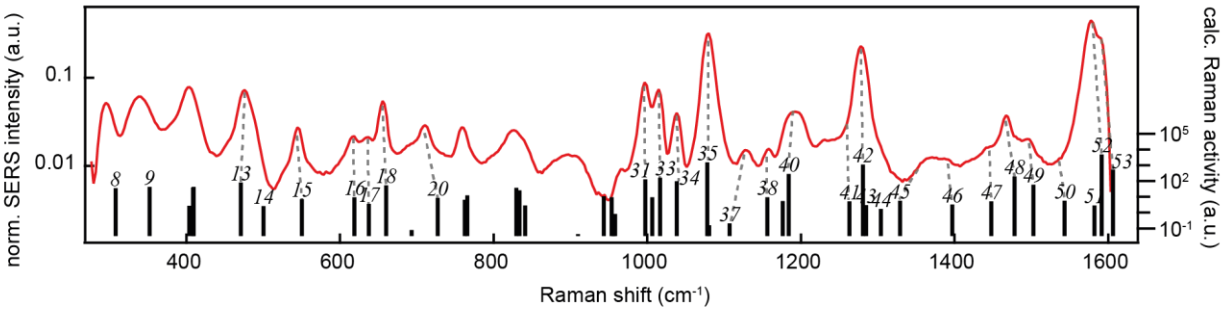

Figure S3: Background-subtracted global nanocavity spectrum and calculated vibrational modes plotted on a log scale to visualise both large and small peaks for tentative peak assignment. A vibrational frequency down-scaling of 0.975 resulted in the best agreement with experimental data, as typical for DFT.

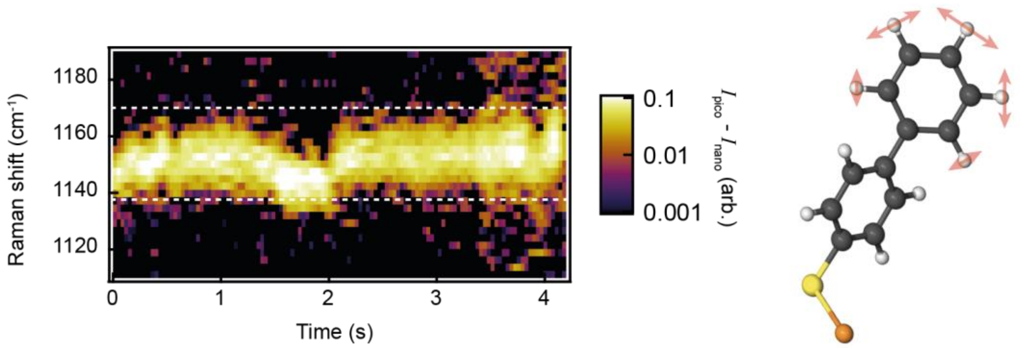

Figure S4: Typical spectral shifts observed within individual time scans, here shown for a picocavity peak assigned to vibrational mode 39 (in-plane wagging motion of hydrogen atoms in upper phenyl group, illustrated on the right), with the upper and lower integration bounds marked with white dashed lines.

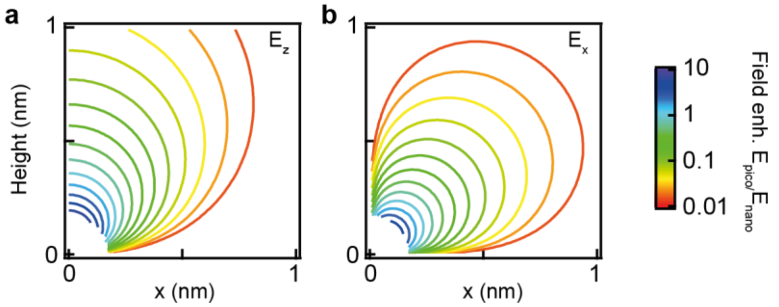

Fig. S5: Analytical approximation for picocavity field, showing the out-of-plane component (a) and in-plane component (b) as introduced in ref. [45] of the main text.[3]

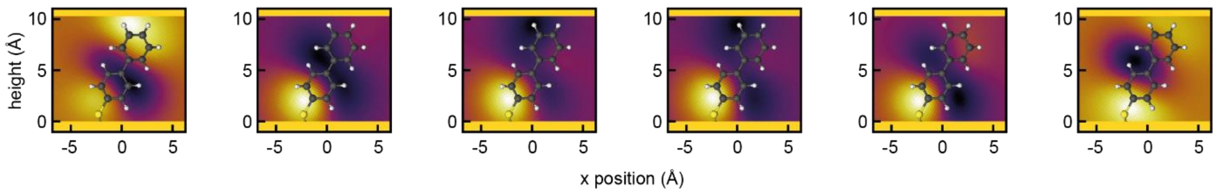

Figure S6: Gradient field maps for Au-Au NPoM sample using commercial NPs showing one NP picocavity type and 5 substrate picocavity types.

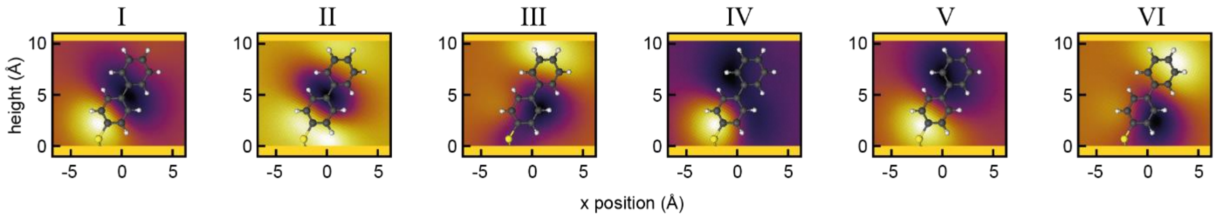

Figure S7: Gradient field maps for Au-Au NPoM sample using homemade NPs showing two NP picocavity types and 4 substrate picocavity types.

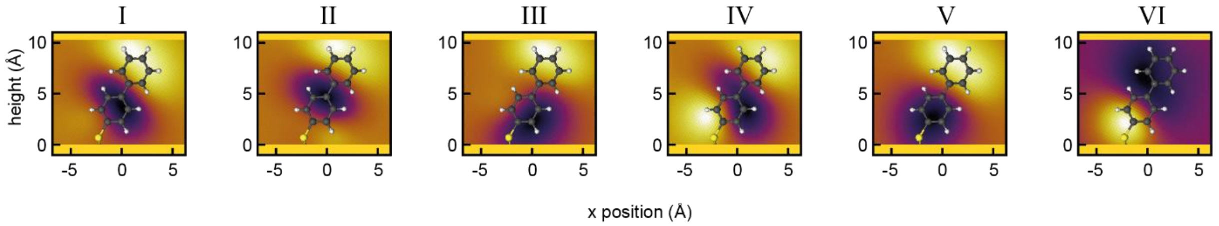

Figure S8: Gradient field maps for Au-Pd/Au NPoM sample using homemade NPs showing four NP picocavity types, 1 substrate picocavity types and 1 mixed type (IV), with the three largest clusters coming from the NP.

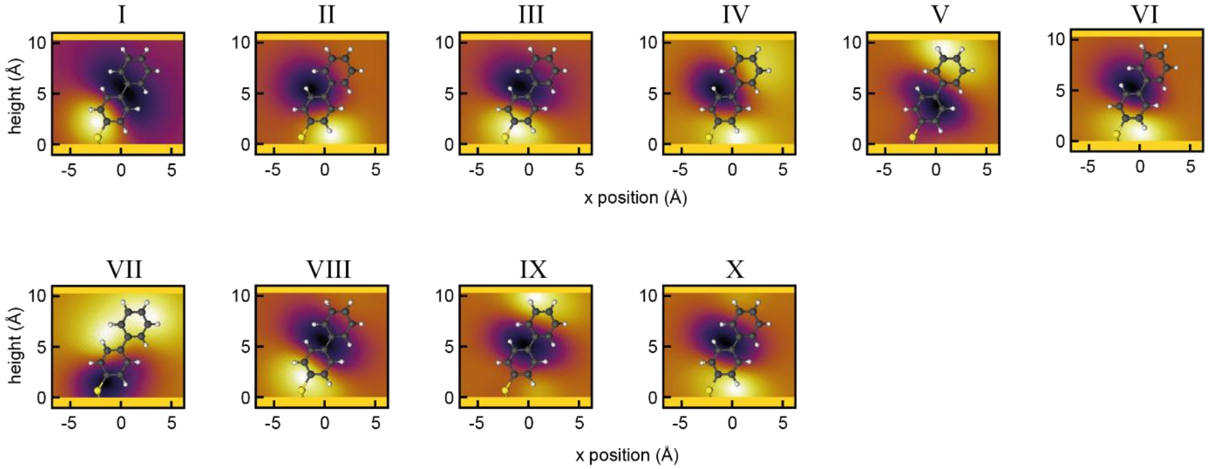

Figure S9: Gradient field maps for Au@Pd-Au NPoM sample using homemade NPs showing three NP picocavity types, and 7 substrate picocavity types, with the three largest clusters coming from the substrate.

AUTHOR INFORMATION

Corresponding Authors

Bart de Nijs, NanoPhotonics Centre, Cavendish Laboratory, University of Cambridge, CB3 0HE Cambridge, UK, orcid.org/0000-0002-8234-723X, Email: bd355@cam.ac.uk

Stuart J. Gibson, School of Physics and Astronomy, Ingram Building, University of Kent, Canterbury, CT2 7NH, UK, orcid.org/0000-0002-7981-241X, Email: s.j.gibson@kent.ac.uk

Authors

Alex D. Poppe, School of Physics and Astronomy, Ingram Building, University of Kent, Canterbury, CT2 7NH, UK.
Jack Griffiths, NanoPhotonics Centre, Cavendish Laboratory, University of Cambridge, CB3 0HE Cambridge, UK
Shu Hu, NanoPhotonics Centre, Cavendish Laboratory, University of Cambridge, CB3 0HE Cambridge, UK
Jeremy Baumberg, NanoPhotonics Centre, Cavendish Laboratory, University of Cambridge, CB3 0HE Cambridge, UK
Margarita Osadchy, Computer Science Department, University of Haifa, Haifa, Israel

Notes

The authors declare no competing interest.

(1) Benz, F.; Chikkaraddy, R.; Salmon, A.; Ohadi, H.; de Nijs, B.; Mertens, J.; Carnegie, C.; Bowman, R. W.; Baumberg, J. J. SERS of Individual Nanoparticles on a Mirror: Size Does Matter, but so Does Shape. *J. Phys. Chem. Lett.* **2016**, *7* (12), 2264–2269.
(2) Nijs, B. de; Bowman, R. W.; Herrmann, L. O.; Benz, F.; Barrow, S. J.; Mertens, J.; Sigle, D. O.; Chikkaraddy, R.; Eiden, A.; Ferrari, A.; Scherman, O. A.; Baumberg, J. J. Unfolding the Contents of Sub-Nm Plasmonic Gaps Using Normalising Plasmon Resonance Spectroscopy. *Faraday Discuss.* **2015**, *178* (0), 185–193.
(3) Zhang, Y.; Dong, Z.-C.; Aizpurua, J. Theoretical Treatment of Single-Molecule Scanning Raman Picoscopy in Strongly Inhomogeneous near Fields. *Journal of Raman Spectroscopy* **2021**, *52* (2), 296–309.
